# 

**Published:** 1917-07-07

**Authors:** 


### CONTENTS.

PAGE

**The Mesopotamia Commission Report...** ... 263

**Physical Exercises from a National Standpoint** ... ... ... ... ... ... ... 264

**Hospital and Institutional News** ... ... ... 265

The Opening of Baby Week—An Annual Baby Week—Hospital Sunday Fund Results—The Dumping of German Wounded—The American Doctors at the Front—The York Workpeople's Fund—Fatal Shaving-brushes—The Shortage of Doctors—Retirement of a Hospital Secretary—Is the Pensions Committee to send Discharged Soldiers to the Workhouse?—No Compulsory Training for Wounded—Disablement and Decentralisation—Medical Aid for Poor Women—The Housing Problem—Chester Children Losing Weight—Notification of Measles—A Soldier's Legacy—What Causes Alcoholic Excess?—The Practice of Dictation—Poor-Law Institutional Treatment and the National Insurance Act—Disagreement of the Officers with the Board—The Fire at the First London—The Lighter Side—Brighton and the Babies—The New Trouble—War Bread.

**Venereal Disease in Scotland** ... ... ... ... 271

The Trend of Progress in Treatment.

**The Federation of Hospital Treatment for Children** ... ... ... ... ... 272

The I.C.A.A. as a Clearing House.

PAGE

**Concerning Woman Suffrage** ... ... ... 273

**The Institutional Library** ... ... ... ... 275

**Guy's Hospital and the Calling-up of Doctors** 277

Correspondence with the Local Government Board.

**Blood Investigations in Flying Men** ... ... 277

**The Editor's Letter-Box** ... ... ... ... 278

British Nurses and Nursing. A Campaign of Misrepresentation and Personal Ambitions.

**The Matrons' and Sisters' Department** ... 279

St. Thomas's Hospital. An Historic Group.

The Position of British Nursing. By Sir Henry Burdett, K.C.B., K.C.V.O.

Round the Hospitals.

The American Nurses at the Front—London Schools of Massage Development—A Step to be Retraced—The College of Nursing Report—First Report of the Scottish Board—A Campaign of Misrepresentation and Personal Ambition.

**The College of Nursing, Limited** ... ... 283

The Second Annual Report of the Council.

**Parliament and the Profession** ... ... ... vi

The Probationer's Training and Time-Table: Answer to May Question.

Substitutes for Potatoes: Answer to May Question.

